# Survivorship care plan utilization in Australia and New Zealand: survivors’, parents’ and healthcare providers’ perspectives

**DOI:** 10.1007/s00520-025-09238-7

**Published:** 2025-02-12

**Authors:** Rebecca E. Hill, Joanna E. Fardell, Rebecca Mercieca-Bebber, Claire E. Wakefield, Christina Signorelli, Kate Webber, Karen A. Johnston, Richard J. Cohn

**Affiliations:** 1 School of Clinical Medicine, UNSW Medicine and Health, UNSW Sydney, Randwick, NSW Australia; 2 Kids Cancer Centre, Behavioural Sciences Unit, Sydney Children’s Hospital, Sydney , NSW Australia; 3 Trials Centre, NHMRC Clinical, The University of Sydney, Sydney, NSW Australia; 4 School of Clinical Sciences at Monash Health, Faculty of Medicine, Monash University, Monash, VIC Australia; 5 Medical Oncology Department, Monash Health, Clayton, VIC Australia

**Keywords:** Cancer, Survivorship, Survivorship care plans, Treatment summary, Survivor

## Abstract

**Purpose:**

As part of survivorship care, many health authorities recommend survivorship care plans (SCPs). The aim of this study was to understand survivors’ SCP receipt and use, clinical/demographic factors associated with use, and providers’ SCP practices.

**Methods:**

We surveyed Australian and New Zealand survivors of adult and childhood cancer (including parent proxies for survivors aged < 16 years). We fitted binomial logistic regression models to examine the relationship between survivors’ clinical and sociodemographic characteristics, and SCP receipt. We also surveyed oncology health providers regarding current SCP provision practices, perceived receipt, and usefulness.

**Results:**

We recruited 1123 cancer survivors (499 adult cancer survivors and 624 childhood cancer survivors, including 222 parent proxies) and 21 healthcare providers. 10.7% of adult and 22.0% of childhood cancer survivors recalled receiving SCPs. SCP receipt was more likely for adult cancer survivors diagnosed with prostate cancer, low-risk cancer diagnoses and older at study participation, and childhood cancer survivors treated with chemotherapy or younger at study participation. Across both groups, a higher level of education attainment was predictive of SCP use. Most healthcare providers estimated that < 15% of adult and > 75% of childhood cancer survivors received SCPs.

**Conclusions:**

Few survivors of adult or childhood cancer reported receiving a SCP, and there were sociodemographic and clinical differences in those who did and did not receive and use their SCP. SCP recipients used and valued them, but healthcare providers indicated potential areas for improvement with SCP provision. Consideration may be needed regarding SCP format, presentation and content.

**Supplementary information:**

The online version contains supplementary material available at 10.1007/s00520-025-09238-7.

## Introduction

Many cancer survivors are at risk of late morbidity and mortality due to their cancer and its treatment [1]. Late effects can be life-threatening or life-altering [2], so follow-up care is recommended [3]. To improve cancer-related follow-up care, the National Academy of Medicine recommended survivorship care plans (SCPs) [4]. SCPs provide information about survivors’ cancer and treatment history, recommended schedule of surveillance and follow-up, and available support services. SCPs are beneficial to survivors’ proximal outcomes, such as awareness of follow-up care [5–7], and to increase healthcare providers knowledge of late effects and follow-up care [8].

However, less than 40% of survivors of adult or childhood cancer receive a SCP [9–12]. Socioeconomic and clinical differences in SCP receipt have been reported among US survivors of adult cancer. Three large-scale studies (N = 740–46,408) found that SCP receipt was most likely among survivors who were diagnosed with breast or colorectal cancer, younger (i.e. aged < 65 years), college-educated, partnered, women, covered by health insurance that paid for their cancer treatment, or had stage I cancer [9, 11, 13]. No significant differences in self-reported SCP receipt were found among Australian survivors of adult cancers [10]. While these studies provide useful insights into SCP receipt, they did not examine whether, or which, survivors actually utilised their SCP. To our knowledge, no study has been conducted to explore differences in SCP provision for survivors of childhood cancer.

It is unclear whether adult or childhood cancer survivors who receive a SCP, actually use them. To understand the broad context of SCP receipt, it is important to capture how SCPs are provided to survivors and whether they align with survivors’ preferences. Healthcare interventions are more effective when designed with stakeholder input [14].

Our study therefore aimed to: (i) understand the relationship between cancer survivors’ (including parent proxies) self-reported SCP receipt and use, and sociodemographic and clinical characteristics; (ii) describe survivors’ (and parent proxies’) SCP delivery and format preferences; and (iii) describe healthcare providers’ SCP creation and distribution practices. Adult and childhood cancer survivors were included as both groups require follow-up care [15].

## Materials and methods

### Participants

#### Survivors

We recruited Australian survivors of any adult cancer who were at least 18 years old and completed curative intent surgery, chemotherapy and/or radiation therapy at least six months before the survey, between April and November 2018. We recruited Australian and New Zealand survivors of childhood cancer who were at least 16 years old, and at least five years post-diagnosis between 2012 and 2018. Childhood cancer survivors included in the study were five or more years from diagnosis, reflecting the eligibility to attend a Survivorship Clinic in Australia and New Zealand at the time of the study. We recruited parents (via self- and proxy-report) for survivors aged below 16 years, given that younger survivors may have difficulty completing our survey. Participants needed to be English-proficient and in remission.

#### Healthcare providers

We recruited Australian and New Zealand healthcare providers, employed within any oncology setting, between August and December 2020.

### Recruitment

#### Survivors

We recruited survivors of adult cancers via two national, online research panels (PathFinder: www.pathfinderregister.com.au; and Register4: www.register4.org.au). PathFinder included 650 men interested in prostate cancer research; and Register4 had 38,758 members (aged: 20–96 years; 96.6% female as it is seed funded by the National Breast Cancer Foundation). PathFinder and Register4 invited members via email and sent one reminder. Our survey was advertised on their websites, allowing for participants to also be self-selected. For eligible survivors of childhood cancer, we searched participating hospitals’ electronic medical records. We emailed or posted our survey to survivors of childhood cancer (or parents) and contacted non-respondents up to four times. Completion of the survey implied consent to participate. Ethics approval was granted by UNSW Sydney (HC180032, HC190101) and the Institutional Review Boards of 11 Australian and New Zealand hospitals (12/POWH/345).

#### Healthcare providers

We recruited healthcare providers via Australian and New Zealand Children’s Haematology/Oncology Group (ANZCHOG), and the Clinical Oncology Society of Australia (COSA) Survivorship Group. These organisations invited their members via email, followed by two reminders. ANZCHOG is a multidisciplinary, non-for-profit organisation for healthcare providers interested in advancing childhood cancer care. The COSA Survivorship Group comprises healthcare professionals interested or employed in cancer survivorship care or research, with an aim to improve outcomes for all cancer survivors in Australia.

### Outcomes

#### Demographic and clinical information

##### Survivors

 We asked for survivors’ (or parents to report their child’s) sex, current age, age at diagnosis, cancer diagnosis, stage, and treatment/s received, and cancer treatment completion date. Survivors and parents also self-reported their education level, marital, employment status, private health insurance status, income, and residential postcode.

##### Healthcare providers

We asked healthcare providers their sex, occupation, employment setting, length, and country, and years working in survivorship care.

#### SCP receipt

##### All participants

We asked survivors whether they recalled ever receiving a SCP and healthcare providers if they gave SCPs to their patients [0 = “no”, 1 = “yes”]. 

##### Survivors

We asked the date they received their SCP.

#### SCP use

##### Survivors

We asked whether they remembered discussing/referring to their SCP, or sharing it with a healthcare provider, their partner, or another party (e.g. employer). We created a dichotomised score for analysis [0 = “no SCP use”; ≥ 1 = “SCP use”]. 

##### Healthcare providers

Using four items adapted from Birken et al. [16], we assessed healthcare providers’ SCP use, and their perception of their colleagues’ and patients’ SCP use (see Supplementary information).

#### SCP dissemination practices

##### Healthcare providers

We used 11 purpose-designed items to determine how, where and by whom SCPs are created and delivered to survivors (see Supplementary information). We assessed healthcare providers’ confidence in providing follow-up care on a three-point Likert scale [1 = low, 2 = adequate, 3 = high], using five items adapted from Smith et al. [17]. Higher scores indicate greater confidence.

#### Usefulness of, and satisfaction with, SCPs

##### Survivors

We assessed the perceived usefulness of SCPs using a five-point Likert scale [1 = “not at all useful” to 5 = “very useful”]. We dichotomised this variable for analysis [“not useful” = 1, 2 or 3; “useful” = 4 or 5]. 

##### Healthcare providers

We assessed whether healthcare providers were satisfied with SCPs provided to patients at their workplace and whether SCPs were useful for patient care [18]. We examined perceived SCP benefits and concerns using 20 items on a five-point Likert scale [1 = strongly disagree to 5 = strongly agree], developed by expert consensus (see Supplementary information).

#### Preferences for SCP format

##### Survivors

We asked participants their preferred SCP format [1 = “online”, 2 = “paper”, 3 = “online and paper version”]. We dichotomised this variable [0 = no (response option 1 or 3), 1 = yes, paper version only (response option 2)].

#### Preferences for timing of SCP receipt

##### Survivors

We asked the ideal time to receive a SCP [adult: 1 = “end of treatment”, 2 = “first referred back to general practitioner”, “3 = other, please specify”; child: 1 = “end of treatment”, 2 = “one year post-treatment”, 3 = “five years post-diagnosis” and 4 = “other”.

### Data analysis

We used IBM SPSS Statistics version 24.0. We separately analysed: (i) survivors of adult, and (ii) childhood cancer, including parents, unless otherwise specified. We compared differences in SCP receipt and use by clinical and sociodemographic factors using chi-squared tests, Fisher’s exact tests (FET), and independent samples *t*-tests.

We fitted binomial logistic regression models to determine whether (i) SCP receipt was predicted by survivors’ clinical characteristics, including cancer history or sociodemographic factors (see Table 3); and (ii) SCP format preferences by age at study participation, sex, education level, income, residential status, or parent-proxy status. Across these three models, regressions required a sample size of 300 and 400 participants for four and six independent variables, respectively [19]. We included covariates used in previous SCP research [20]. We deemed *p* ≤ 0.05 to be significant.

## Results

### Survivor and parent characteristics

Our study included 1123 individuals: 499 adult and 624 childhood cancer survivors (including 222 parent proxies; see Table 1) Response rate for survivors of adult cancer could not be calculated due to our recruitment approach. Our response rate for survivors of childhood cancer was 53% (624/1,176).

**Table 1:** Participants’ clinical and sociodemographic characteristics and relationships to self-reported SCP receipt and use, *N *= 1123

	Adult cancer survivors (*N *= 499)						Childhood cancer survivors (*N *= 624, including parent proxies)					
	No SCP receipt	SCP receipt	*P* value	No SCP use	SCP use	*P* value	No SCP receipt	SCP receipt	*P* value	No SCP use	SCP use	*P* value
Survivor characteristics; mean (SD)												
Age at study (years)	62.0 (9.9)	65.9 (9.1)	**0.006**	64.7 (10.6)	66.0 (9.0)	0.733	22.1 (9.2)	18.8 (6.6)	** < 0.001**	20.1 (4.9)	18.8 (7.0)	0.365
Age at diagnosis (years)	54.9 (11.3)	60.0 (9.2)	**0.002**	56.1 (12.8)	60.6 (8.6)	0.244	5.6 (4.6)	5.1 (4.2)	0.192	5.9 (5.1)	4.9 (4.1)	0.276
Time since treatment (years)	6.5 (6.7)	5.2 (5.0)	0.226	5.8 (8.2)	5.0 (4.4)	0.710	14.4 (8.1)	11.9 (5.7)	** < 0.001**	11.9 (5.1)	12.2 (6.0)	0.813
Survivor characteristics; *n*												
Sex												
Male	104	24	** < 0.001**	3	21	1.000	225	68	0.543	15	47	0.378
Female	339	29		4	24		246	66		11	51	
Adult cancer diagnosis												
Breast cancer	256	23	** < 0.001**	4	18	0.438	−	−	−	−	−	−
Prostate cancer	89	24		3	21	1.000	−	−	−	−	−	−
Other	99	6		0	6		−	−		−	−	
Childhood cancer diagnosis												
Acute Lymphoblastic Leukaemia	−	−	−	−	−	−	181	50	0.801	11	37	0.672
Other	−	−		−	−		289	84		15	61	
Cancer stage												
Low-risk (Stage 0–II)	201	29	**0.037**	5	23	0.569	−	−	−	−	−	−
Stage III	78	5		0	5		−	−		−	−	
Stage IV	30	0		0	0							
Chemotherapy												
No	232	39	**0.003**	4	35	0.347	38	2	**0.005**	1	1	**0.361**
Yes	212	14		3	10		426	128		23	95	
Radiotherapy												
No	185	27	0.197	3	24	0.698	242	68	0.940	10	34	0.673
Yes	259	26		4	21		188	52		13	54	
Surgery												
No	35	4	0.932	1	3	0.450	222	61	0.673	11	47	0.743
Yes	409	49		6	42		207	62		12	44	
Residential location												
Non-metropolitan	152	16	0.643	3	13	0.666	104	24	0.653	1	21	0.040*
Metropolitan	287	35		4	30		332	86		20	62	
Pediatric subgroup												
Survivor	−	−	−	−	−	−	313	81	0.232	23	56	0.003*
Parent	−	−		−	−		161	53		3	42	
Survivor or parent characteristics, *n*												
Education												
Secondary school	87	13	0.411	4	8	**0.041**	162	58	0.083	16	38	**0.037**
Post-secondary	354	40		3	37		300	76		10	60	
Employment												
Not employed	230	32	0.209	4	12	0.208	116	45	**0.043**	14	28	**0.017**
Employed	172	16		3	28		348	88		12	69	
Household income												
< $60,000 p.a. ^a^	164	25	0.304	4	20	1.000	202	55	0.681	16	36	**0.020**
> $60,000 p.a	215	24		3	21		202	60		7	49	
Marital status												
Not partnered	102	11	0.697	3	8	0.154	251	77	0.405	21	53	**0.014**
Partnered	339	42		4	37		219	57		5	45	
Private health insurance												
No (Medicare only)	72	10	0.633	4	39	0.090	150	52	0.130	8	40	0.399
Yes	370	43		3	6		314	80		17	57	

^a^*P.A.*, per annum

^b^Cancer stage is not available for acute lymphoblastic leukemia survivors (as tumours generally do not develop) so we excluded these participants

^c^Many participants received multiple treatment modalities

^d^ “Other treatments” included cryotherapy, immunotherapy (e.g. monoclonal antibodies), retinoid therapy and steroids

^e^ “Other haematopoietic cancers” included acute myeloid leukemia, Hodgkin’s lymphoma and Non-Hodgkin lymphoma; Bold = *p* ≤ 0.05

Survivors of adult cancer were, on average, aged 62.4 years at study participation (range: 28–82 years) and 21.3 years for survivors of childhood cancer (range: 7–61 years). Prostate cancer survivors were significantly older than survivors of other adult cancers ((M = 67.6, SD = 7.8 vs. M = 60.9, SD = 10.0), t(497) =  < 0.001). Most participants were female (adult: 74.2%, 369/497; child: 51.5%, 320/621) and had post-high school qualifications (adult: 79.6%, 395/496; child: 55.9%, 219/392; parent: 75.3%, 165/219). Adults were most commonly diagnosed with breast cancer (56.1%, 280/499) and children with Acute Lymphoblastic Leukaemia (38.5%, 237/620).

### Healthcare provider characteristics

We recruited 21 healthcare providers (Table 2), who were mostly employed within hospital settings (95.2%, 20/21) in metropolitan areas (90.5%, 19/21). Most (66.7%, 14/21) worked in adult oncology, 23.8% (5/21) in pediatric oncology, and 9.5% (2/21) worked across both populations. On average, providers had worked in survivorship care for 10.4 years (range: 1.5–35 years). Most were employed as medical oncologists (38.1%, 8/21) or nurses (38.1%, 8/21) for a mean of 14.5 years (range: 3–35 years).

**Table 2:** Healthcare provider characteristics, *N* = 21

Healthcare provider characteristics	No	%
Female	16	76.2
Australian	20	95.2
Occupation		
Nurse^a^	8	38.1
Pediatrics	4	19.0
Young adult or adult	5	23.8
Medical oncologist	8	38.1
Pediatric oncologist	2	0.1
Radiation oncologist	1	0.05
Dietitian	1	0.05
Oncology pharmacist	1	0.05
Place of employment^a^		
Public hospital	21	100.0
Private hospital	3	14.3
Community health centre	1	0.05
University	2	0.1
Area of employment^a^		
Metropolitan	19	90.5
Rural	4	19.0
Remote	1	0.05
Cancer patient population^a^		
Childhood	6	28.6
Adolescent	7	33.3
Young adult	8	38.1
Adult	16	76.2
	Mean (SD)	Range
Time in current role (years)	14.5 (9.5)	3–35
Time within oncology (years)	17.7 (8.9)	4–40

^a^Multiple options may have been selected

### Survivors’ SCP receipt

**Fig. 1:**
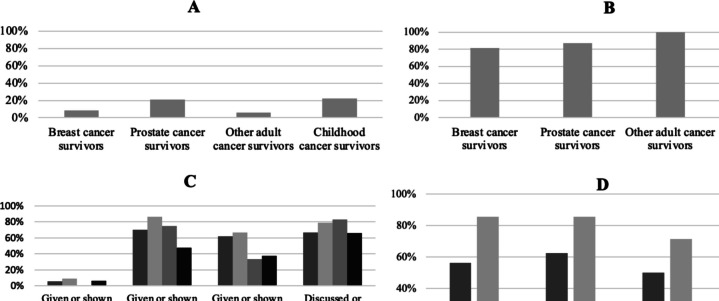
(**A**) Proportion of cancer survivors that reported receiving a survivorship care plan (SCP). **B** Proportion of cancer survivors that reported using their SCP. **C** Proportion of cancer survivors that endorsed statements regarding how they utilised their SCP. **D** Proportion of healthcare providers in adult and pediatric oncology that created, delivered, used, or received a patient’s SCP

Few participants recalled receiving a SCP (10.7% of adult and 22.0% of childhood cancer survivors; Fig. 1). Survivors of breast, prostate and other adult cancers reported receiving their SCP, on average, 1.6, 0.5 and 0.0 years following cancer treatment completion (total adult range: 0.0–10.0 years); whereas it was 9.4 years for survivors of childhood cancer and parents (range: 1.2–22.9 years). Parents reported receiving their child’s SCP significantly earlier than survivors of childhood cancer, *t *(44) = 3.054, *p* = 0.002 (M = 6.5, SD = 3.6 vs M = 10.7, SD = 4.7 years post-treatment).

After controlling for covariates, we found survivors of adult cancer who received a SCP, as compared to non-recipients, were significantly more likely to: (i) be diagnosed with prostate cancer (OR 2.763, 95%CI 1.272–6.003, *p* = 0.010); (ii) have a low-risk cancer stage (i.e., stage 0–II) (OR 0.305, 95%CI 0.108–0.861, *p* = 0.025); or (iii) be older at study participation (OR 1.051, 95%CI 1.012–1.091, *p* = 0.009). Survivors of childhood cancer who received a SCP, as compared to non-recipients, were more likely to be: (i) treated with chemotherapy (OR 10.814, 95%CI 1.456–80.297, *p* = 0.020); or (ii) younger at study participation (OR 0.968, 95%CI 0.939–0.998, *p* = 0.037). SCP receipt was not associated with any other cancer history or sociodemographic variables collected in this study (see Table 3).

**Table 3:** Regressions of survivors’ and parents’ SCP receipt and format preferences by cancer history and sociodemographic factors

SCP receipt factors	Survivors of adult cancer (*N *= 493)				Survivors of childhood cancer and parents (*N* = 562)			
1. Cancer history	OR	95% CI lower	95% CI upper	*p*	OR	95% CI lower	95% CI upper	*p*
Age at diagnosis	1.031	0.997	1.066	0.072	0.970	0.922	1.021	0.245
Disease site (ref: breast)								
ALL	−	−	−	−	0.894	0.590	1.354	0.595
Prostate	2.763	1.272	6.003	0.010*	−	−	−	−
Other adult cancer	0.747	0.384	1.965	0.555	−	−	−	−
Cancer stage (ref: low risk)								
Advanced (stage III-IV)	0.305	0.108	0.816	0.025*	−	−	−	−
Unknown stage	0.633	0.313	1.279	0.203	−	−	−	−
Chemotherapy (ref: no)								
Yes	0.912	0.424	1.961	0.814	10.814	1.456	80.297	0.020*
Pediatric subgroup (ref: survivor)								
Parent	−	−	−	−	0.982	0.620	1.555	0.938
2. Sociodemographic	(*N* = 426)				(*N* = 450)			
Age at study participation	1.051	1.012	1.091	0.009*	0.968	0.939	0.998	0.037*
Education level (ref: secondary school)								
Post-secondary school	0.723	0.356	1.468	0.369	0.845	0.516	1.384	0.504
Income (ref: < $60,000)								
> $60,000	1.159	0.585	2.297	0.673	1.035	0.631	1.699	0.892
SCP receipt factors	Survivors of adult cancer (*N* = 419)				Survivors of childhood cancer and parents (*N* = 411)			
2. Sociodemographic	OR	95% CI lower	95% CI upper	*p*	OR	95% CI lower	95% CI upper	*p*
Private health insurance status (ref: no)								
Yes	0.819	0.373	1.798	0.618	0.666	0.407	1.089	0.105
Residential location (ref: non-metropolitan)								
Metropolitan	0.959	0.503	1.828	0.898	1.279	0.732	2.235	0.388
3. SCP format preferences	OR	95% CI lower	95% CI upper	*p*	OR	95% CI lower	95% CI upper	*p*
Age at study participation	1.065	1.035	1.095	< 0.001*	1.025	0.979	1.073	0.296
Sex (ref: male)								
Female	1.537	0.913	2.587	0.106	1.053	0.625	1.772	0.847
Education level (ref: secondary school)								
Post-secondary school	1.314	0.75	2.304	0.340	0.387	0.224	0.668	< 0.001*
Income (ref: < $60,000)								
> $60,000	1.05	0.647	1.706	0.842	0.693	0.389	1.233	0.212
Residential location (ref: non-metropolitan)								
Metropolitan	1.237	0.779	1.963	0.368	0.974	0.539	1.759	0.931
Pediatric subgroup (ref: survivor)								
Parent	−	−	−	−	2.394	1.007	5.692	0.048*

*SCP*, survivorship care plan; *OR*, odds ratio; *95% CI*, 95% confidence interval; * = *p* ≤ 0.05

### Survivors’ SCP use

Of participants who reported receiving a SCP, 86.5% of adult (45/52) and 79.0% of childhood cancer survivors reported using their SCP (98/124; Fig. 1). Most participants reported that their SCP was useful (breast: 63.2%, 12/19; prostate: 81.3%, 13/16; other adult: 57.1%, 4/7; child: 61.9%, 73/118).

Both survivors of adult and childhood cancer (and parent proxies) were more likely to use their SCP if they had higher levels of educational attainment (adult: *p* = 0.041, FET; child: χ^2^(1, N = 124) = 4.331, *p* = 0.037, Table 1). SCP use was not associated with any other variable for survivors of adult cancer. Survivors of childhood cancer were more likely to use their SCP if they were: (i) employed (χ^2^(1, N = 123) = 5.690, *p* = 0.017); (ii) earning above $60,000AUD per annum (p.a.) (χ^2^(1, N = 108) = 5.369, *p* = 0.020); (iii) living in a non-metropolitan area (*p* = 0.040, FET); (iv) partnered (χ^2^(1, N = 124) = 6.082, *p* = 0.014); or (v) parents, rather than survivors (χ^2^(1, N = 124) = 8.717, *p* = 0.003).

### Survivors’ preferences for SCP timing and format

Most participants reported that they would like to receive their SCP at the end of treatment (breast: 69.3%, 192/277; prostate: 50.4%, 57/113; other adult: 62.9%, 66/105; child: 53.5%, 302/564), in both paper and electronic format (breast: 60.7%, 167/275; prostate: 55.9%, 62/111; other adult: 60.8%, 62/102; child: 71.8%, 392/546). Some participants preferred solely a SCP that was electronic (breast: 10.9%, 30/275; prostate: 14.4%, 16/111; other adult: 11.8%, 12/102; child: 8.4%, 46/546) or paper-based (breast: 28.4%, 78/275; prostate: 29.7%, 33/111; other adult: 27.5%, 28/102; child: 19.8%, 108/546).

After controlling for covariates, the following groups were significantly more likely to prefer paper-based SCPs to other formats: (i) survivors of adult cancer who were older at study participation (OR 1.065, 95%CI 1.035–1.095, *p* < 0.001); (ii) survivors of childhood cancer with lower levels of education attainment (OR 0.387, 95%CI 0.224–0.668, *p* < 0.001); or (ii) parents, rather than childhood cancer survivors (OR 2.394, 95%CI 1.007–5.692, *p* = 0.048; see Table 3).

### Healthcare providers’ creation and dissemination of SCPs

#### SCP design and timing

When asked how SCPs were produced, most healthcare providers indicated nurses (adult: 63.6%, 7/11; child: 50%, 3/6) used a SCP template designed by their workplace (adult: 62.5%, 5/8; child: 66.7%, 4/6). Healthcare providers reported that most often paper-based SCPs (70%, 7/10) were given to survivors of adult cancer at the end of treatment (41.7%, 5/12); survivors of childhood cancer most often received a combined paper- and electronic-based SCP (66.7%, 4/6) five years post-treatment (85.7%, 6/7).

#### SCP delivery

Most adult oncology providers estimated that at their workplace SCPs were received by 0–15% of survivors (75.0%, 9/12) or their primary care providers (72.7%, 8/11). Pediatric healthcare providers (71.4% of those surveyed, 5/7) estimated over 75% of patients or their primary care providers received SCPs (42.9%, 3/7). Survivors and/or children’s families were reported being given their SCPs from hospital nursing staff (adult: 43.8%, 7/16; child: 85.7%, 6/7) and during in-person consultations (adult: 44.4%, 4/9; child: 83.3%, 5/6). General information was often supplied about follow-up care (adult: 56.3%, 9/16; child: 71.4%, 5/7) and a letter addressed to primary care providers (adult: 62.5%, 10/16; child: 71.4%, 5/7). Some survivors were reported to be offered nurse-led (adult: 43.8%, 7/16; child: 71.4%, 5/7) and/or oncologist-led counselling about their future care/health (adult: 31.3%, 5/16; child: 85.7%, 6/7).

#### Satisfaction with, and perceived benefits of, SCPs

**Fig. 2:**
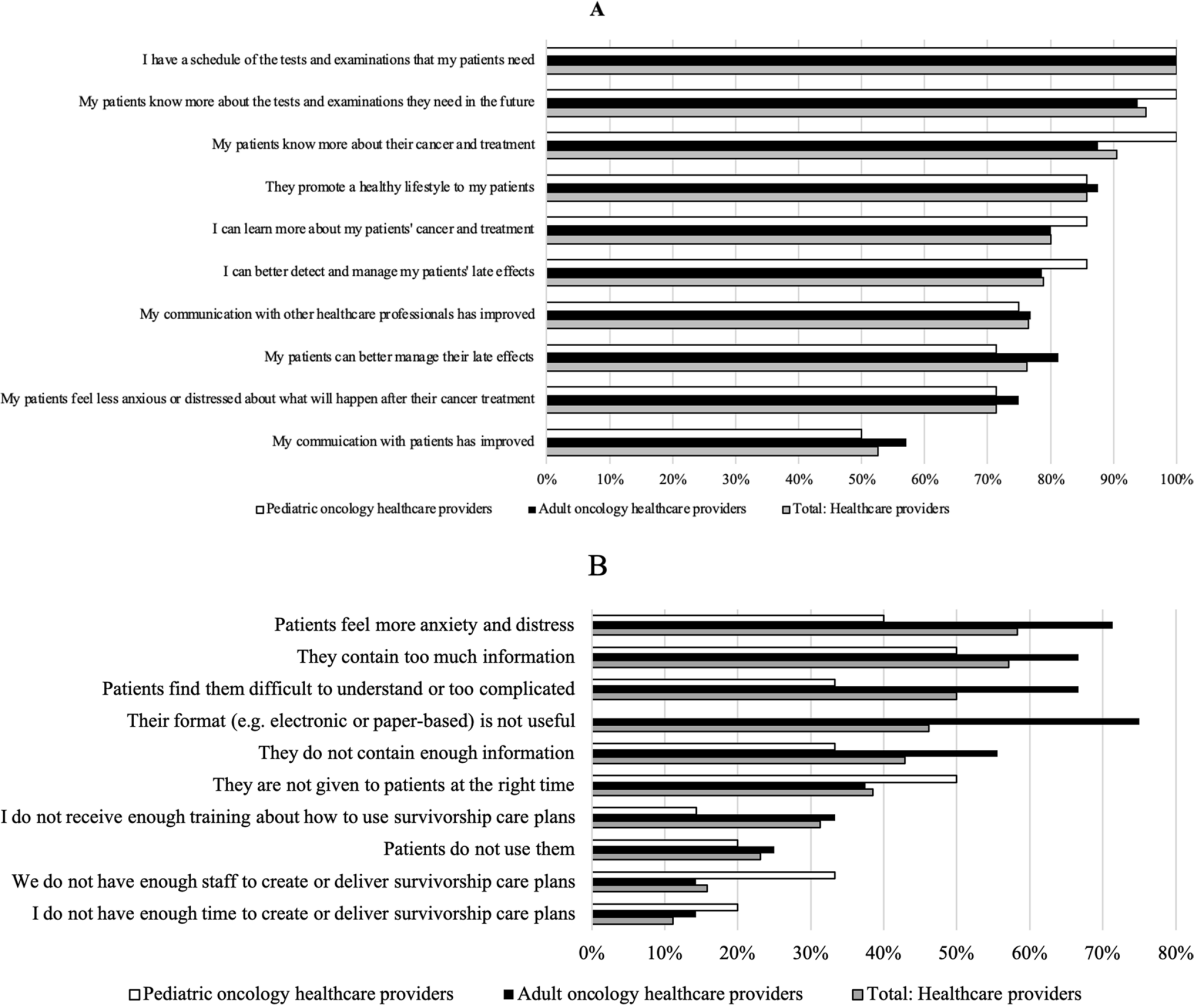
(**A**) Proportion of healthcare providers that endorsed statements regarding perceived benefits of survivorship care plans. **B **Proportion of healthcare providers that endorsed statements regarding perceived concerns about survivorship care plans. Note. No pediatric oncology healthcare providers endorsed the statement “Their format (e.g. electronic or paper-based) is not useful

Many healthcare providers were satisfied with SCPs (adult: 60%, 9/15; child: 71.4%, 5/7), and found SCPs useful for patient care (adult: 71.4%, 10/14; child: 100%, 7/7). Adult and pediatric healthcare providers, respectively, endorsed 7.6 and 7.8/10 benefits of SCPs to survivors or their interaction with them (see Fig. 2). All participants agreed having a “schedule of tests and examinations” for patients was useful (100.0%, 18/18).

#### Perceived SCP concerns

Adult and pediatric healthcare providers endorsed a mean of 4.6 and 2.8/10 concerns respectively about the content, production, or effect of SCPs on survivors (see Fig. 2). The most frequent concern reported across providers was that SCPs cause “patients [to] feel more anxiety and distress” (total: 58.3%, 7/12; adult: 71.4%, 5/7; child: 40%, 2/5); followed by SCPs “contain too much information” (total: 57.1%, 8/14; adult: 66.7%, 6/9; child: 50%, 3/6). Many adult oncology providers reported there was a lack of support or resources (i.e., training, information sessions or SCP templates) for employees wishing to provide SCPs to survivors at their workplace (71.4%, 10/14).

#### Confidence in providing follow-up care

Most healthcare providers reported a high level of confidence in their ability to: (i) screen for cancer recurrence (adult: 100%, 14/14; child: 100%, 7/7); (ii) manage survivors’ anxiety/fear of cancer recurrence (adult: 92.9%, 13/14; child: 85.7%, 6/7); (iii) provide information on late effects (adult: 93.3%, 14/15, child: 100%, 7/7); (iv) recommend surveillance/screening for late effects (adult: 85.7%, 12/14; child: 100%, 7/7); and (v) advise on healthy behaviours/lifestyles (adult: 100%, 16/16; child: 100%, 7/7).

## Discussion

We sought to provide insights into the receipt and utilisation of survivorship care plans (SCPs) among cancer survivors in Australia and New Zealand and to examine how our observations could provide impetus for policy and procedural change to benefit survivors. Our results suggest that cancer survivors and healthcare providers valued SCPs, but few survivors recalled receiving them. Most SCP recipients used their SCPs. Self-reported SCP receipt was higher among low-risk cancer stage (i.e., stage 0–II) or older survivors of adult cancer, and younger or chemotherapy treated survivors of childhood cancer. A higher level of education attainment was predictive of SCP use across both groups of survivors. Surveyed healthcare providers estimated that < 15% of survivors of adult cancer, and > 75% of childhood cancer survivors, received SCPs. Healthcare providers also identified health system level concerns, which may hinder the incorporation of SCPs into routine care.

Consistent with our results, previous studies reported that only 10–40% of survivors of adult cancer recall receiving their SCP, even where provision was confirmed by medical records [21–23]. Over time survivors may experience a decline in their recall of follow-up information [24], possibly explained by memory problems, low health literacy or limitations with SCP provision [25]. In our study adult oncology providers reported a lack of support and resources for SCP implementation, corroborating published research [6, 16, 26]. As has previously been documented, some providers, across adult or pediatric oncology, also indicated insufficient staffing or time for SCP provision [6, 16, 26].

Published studies suggest there are clinical and sociodemographic differences in SCP receipt [9, 11, 13]. Survivors of adult cancer in our study with a low-risk cancer stage were more likely to report SCP receipt [13, 27]. Survivors with advanced cancer may be less likely to receive a SCP because they were not discharged to survivorship care or self-selected not to participate in research [28]. We did not find any difference in SCP receipt due to socioeconomic factors for survivors. Australian and New Zealand survivors have access to healthcare under universal healthcare, which may reduce inequalities in SCP receipt attributable to a lack of health insurance reported in the US study by Timsina et al. [11]. Prostate cancer survivors in our study also had greater self-reported SCP receipt, but this must be treated with caution, given our recruitment from the PathFinder registry, which may over-represent engaged and health literate survivors.

We found older survivors of adult cancer and younger survivors of childhood cancer were more likely to recall receiving a SCP. This finding is inconsistent with US studies in which SCP receipt was greater for younger survivors of adult cancer [11, 13]. It may reflect practices by healthcare providers working with prostate cancer survivors in our study. As above, recency bias or memory issues may have impacted our result for childhood cancer survivors. Some older survivors of childhood cancer in our sample also completed treatment prior to SCPs being recommended in 2006 [4]. Younger survivors of childhood cancer were also more likely to be treated with chemotherapy, which separately predicted SCP receipt; perhaps reflecting the risk of late complications and need for ongoing follow-up from this treatment modality [29].

Self-reported SCP use was high among those who recalled receiving a SCP in our study. Most participants referred to or shared their SCP with their healthcare providers or partner. In adult and childhood cancer survivors, we found that higher educational attainment predicted self-reported SCP use. Education may act as a proxy for health literacy [30]. Some healthcare providers in our study reported that patients may find SCPs difficult to understand. Despite recommendations that health materials be written at a 4th–6th grade level, many SCPs require a reading level between the 10th and 12th grade [31]. Some surveyed healthcare providers indicated that SCPs contain too much information, further contributing to the need for higher levels of health literacy to use SCPs.

Unemployed and lower income survivors of childhood cancer (i.e., earning < $60,000 p.a.) were less likely to report using their SCP, supporting research that vulnerable survivors may experience additional challenges accessing and utilising care [11, 20]. We found SCP use was greatest for survivors living in non-metropolitan areas. These survivors may receive more follow-up care outside of their tertiary centre, which are usually based in metropolitan areas [32]. SCPs may play an important role in communicating survivors’ cancer history or follow-up care needs to healthcare providers. Being a parent, rather than survivor, was also predictive of SCP use; highlighting the importance of SCPs being discussed with survivors as they age. Survivors of childhood cancer, due to their age at treatment, may otherwise be dependent on their parents to relay cancer-related information, including SCPs [33].

Surveyed healthcare providers found SCPs beneficial. All healthcare providers in our study valued having a schedule of recommended tests for their patients, demonstrating how SCPs act as a reference tool [34, 35]. Many reported SCPs improved their communication with patients and other healthcare providers, corroborating the literature [36, 37]. However, some healthcare providers, particularly within adult oncology, reported that SCPs caused patients to “feel more anxiety and distress”. Further training may be needed as our previous meta-analysis found no difference in self-reported anxiety between SCP recipients and non-recipients [8].

Our survey had several limitations. SCP receipt and use were self-reported, subject to recall bias, and SCPs may not have been comparable. Our data did not allow us to establish whether survivors’ long-term health management included actions arising because of advice contained in SCPs or whether SCPs acted as a facilitator for discussions with health-care providers, both of which may be beneficial. Despite being a large multi-centre study, our results may not be generalisable. Breast and prostate cancer survivors were over-represented. Disadvantaged groups, including culturally and linguistically diverse survivors, were underrepresented. Survivors’ SCP format preferences may have changed with the growing use of technology [38].

Our sample of healthcare providers included only healthcare providers working in oncology in a hospital setting and may not be representative. Future SCP research may benefit from studying healthcare providers who may use SCPs to inform survivorship and other medical care for survivors, including primary care or other non-oncology specialist providers. Healthcare providers also did not have the option to indicate examples of when SCPs would or would not be provided. Lastly, we did not contemporaneously recruit healthcare providers and survivors. This time difference may have impacted reported experiences with SCPs, particularly as healthcare providers were recruited during the COVID-19 pandemic when the healthcare system was overburdened.

## Conclusion

Our study assessed cancer survivors’ and parents’ self-reported SCP receipt and use, and healthcare providers’ provision of SCPs. Our findings support existing evidence on the low rates of SCP receipt within survivor populations and contribute to the impetus for policy or procedural changes in the field. This is particularly important as most recipients used their SCP and reported that it was useful; however, survivors and parents with lower levels of educational attainment were less likely to report using their SCP. Revisiting the format, presentation, and content of SCPs, may be important for maximising SCP utilisation. Healthcare providers valued SCPs as a reference tool for their patients’ cancer history and follow-up care requirements. Healthcare providers’ concerns, regarding a lack of training and resourcing in some centres to provide SCPs and SCPs potentially inducing anxiety in their patients, must be addressed.

## Supplementary information

Below is the link to the electronic supplementary material.

ESM 1 (DOCX 36.8)

## Data Availability

The datasets generated and analyzed during the current study are available from the corresponding author on reasonable request.
