# Supplementary material for: Survivorship care plan utilization in Australia and New Zealand: survivors’, parents’ and healthcare providers’ perspectives
Source: Support Care Cancer. 2025 Feb 12;33(3):182. doi: 10.1007/s00520-025-09238-7 (PMC11821783; doi:10.1007/s00520-025-09238-7)
Supplement: Supplementary file 1 — (DOCX 36.8) [file 520_2025_9238_MOESM1_ESM.docx]

Survivorship Care Plan Utilization in Australia and New Zealand: Survivors’, Parents’ and Healthcare Providers’ Perspectives

Rebecca E. Hill, B.Psych^1,2^; Joanna E. Fardell, PhD^1,2^; Rebecca Mercieca-Bebber PhD^3^; Claire E. Wakefield, PhD, MPH^1,2^; Christina Signorelli, PhD^1,2^; Kate Webber, PhD, FRACP, B.Sc(Med) MBBS^4,5^; Karen A. Johnston^1,2^; and Richard J. Cohn, MBBCh, FRACP^1,2^.

^1^School of Clinical Medicine, UNSW Medicine and Health, UNSW Sydney, Randwick, NSW, Australia.

^2^Behavioural Sciences Unit, Kids Cancer Centre, Sydney Children’s Hospital, NSW, Australia.

^3^NHMRC Clinical Trials Centre, The University of Sydney, NSW, Australia.

^4^School of Clinical Sciences at Monash Health, Faculty of Medicine, Monash University, VIC, Australia.

^5^Medical Oncology Department, Monash Health, Clayton, VIC, Australia.

**Correspondence:** Rebecca Hill, Kids Cancer Centre, Kids Cancer Centre, Sydney Children’s Hospital, NSW, Australia. Telephone : +61 2 9382 5556, email: [r.hill@student.unsw.edu.au](mailto:r.hill@student.unsw.edu.au).

Submitted to Supportive Care in Cancer

**SUPPLEMENTARY MATERIAL**

**Healthcare providers’ survivorship care plan use**

1. **Have you ever used/referred to a patient’s a survivorship care plan?**

*A survivorship care plan provides a summary of a patient’s cancer treatment and recommendations for their follow-up care. Also known as the Passport for Care or Survivorship Passport (SurPass).*

- Yes
- No
- Don’t know

1. **Approximately what percentage of your colleagues, who work in childhood cancer survivorship, use/refer to patients’ survivorship care plans?**

- 0-25%
- 26-50%
- 51-75%
- 76-100%
- Don’t know

1. **Approximately what percentage of patients at your workplace receive a survivorship care plan?**

- 0-25%
- 26-50%
- 51-75%
- 76-100%
- Don’t know

1. **Approximately what percentage of patients’ primary care providers receive a survivorship care plan?**

- 0-25%
- 26-50%
- 51-75%
- 76-100%
- Don’t know

**Healthcare providers’ survivorship care plan practices**

1. **What other documentation is provided to patients following their cancer treatment at your centre? Select all that apply.**

- Cover letter about survivorship care
- Letter addressed to their general practitioner/family doctor
- Facing forward: life after cancer treatment (National Cancer Institute)
- General information about survivorship care
- HealthLinks
- OncoLife website: <https://oncolife.oncolink.org/>
- Resources from the Lance Armstrong Foundation on overall health
- Resources from the Children’s Cancer Institute Resources
- Other. Please specify: _______________________________
- No information is provided

1. **What is the format of survivorship care plans at your workplace? Select all that apply.**

- Electronic (e.g. Word document, PDF, link to a website)
- Paper-based
- Other. Please specify: _______________________________

1. **Who receives the survivorship care plan? Select all that apply.**

- Patient
- Patient’s family/friends/caregiver
- Hospital staff: medical
- Hospital staff: nursing
- Medical specialist outside of the hospital
- Primary care provider/family doctor/general practitioner
- Other. Please specify: ___________________________

1. **When do patients receive their survivorship care plan?**

- End of treatment
- 6 months after treatment ends
- 1 year after treatment ends
- 5 years after treatment ends
- Other. Please specify: ___________________________

1. **How are patients given their survivorship care plan? Select all that apply.**

- In person
- Via mail/post
- Via email
- Via a personalised link to a website with the cancer survivorship care plan
- Via a mobile, tablet or iPad application
- Other. Please specify: ___________________________

1. **Who gives patients their survivorship care plan? Select all that apply.**
   - Hospital staff: medical
   - Hospital staff: nursing
   - Medical specialist outside of the hospital
   - Primary care provider/family doctor/general practitioner
   - Other. Please specify: _____________________________
2. **Do patients receive any additional services or support along with their survivorship care plan? Select all that apply.**
   - Yes: counselling with a nurse about their future care and health. Please specify how often or how many times this occurs: _____________________________
   - Yes: counselling with an oncologist about their future care and health. Please specify how often or how many times this occurs: _____________________________
   - Yes: counselling with a psychologist or social worker about their future care and health. Please specify how often or how many times this occurs: _____________________________
   - Yes: follow-up calls about their future care and health. Please specify how often or how many times this occurs: _____________________________
   - Yes: other. Please specify how often or how many times this occurs: _____________________________
   - No
3. **How are survivorship care plans created at your workplace? Select all that apply.**

- Somebody fills in a template created by the hospital
- Somebody fills in a templated created by an external organisation. Please specify which template is used (e.g. ASCO): ___________________________
- Automatically generated using a program. Please specify which program is used: ___________________________
- Other. Please specify: ___________________________
- Not sure

1. **Who usually completes survivorship care plans at your workplace? Select all that apply.**

- Nurse
- Medical oncologist
- Haematologist oncologist
- Radiation oncologist
- Paediatrician
- Cardiologist
- Dietician
- Endocrinologist
- Neurologist
- Radiologist
- Researcher
- Surgeon
- Urologist
- Other. Please specify: ___________________________

1. **What support or resources are available to healthcare professionals who want to use survivorship care plans at your workplace? Select all that apply.**

- No support or resources provided
- Information session
- Manual or information booklet
- Training provided by supervisor or colleague
- Template of cancer survivorship care plan provided
- Other. Please specify: ___________________________

1. **In your opinion, what is the purpose of a survivorship care plan?**

___________________________________________________________________________

___________________________________________________________________________

___________________________________________________________________________

**Healthcare providers’ confidence providing follow-up care**

We would like to know more about your experience and confidence with providing survivorship care for cancer survivors.

Survivorship care involves monitoring patients’ overall health, managing any side/late effects from cancer or its treatment, and checking to see if the cancer has come back.

1. **For patients receiving cancer survivorship care, what is your confidence managing the following?**

|  |  | **Low** | **Adequate** | **High** | **N/A** |
| --- | --- | --- | --- | --- | --- |
|  | Screening for cancer recurrence | **□** | **□** | **□** | **□** |
|  | Anxiety/fear of recurrence | **□** | **□** | **□** | **□** |
|  | Providing information on late effects | **□** | **□** | **□** | **□** |
|  | Providing surveillance/screening for late effects | **□** | **□** | **□** | **□** |
|  | Providing advice on healthy behaviours/lifestyles (e.g. exercise, diet, smoking, etc) | **□** | **□** | **□** | **□** |

**Healthcare providers’ perceived benefits of survivorship care plans**

1. **Please indicate how much you agree with the following statements. Survivorship care plans are useful as:**

|  |  | **Strongly agree** | **Agree** | **Neither** | **Disagree** | **Strongly disagree** | **N/A** |
| --- | --- | --- | --- | --- | --- | --- | --- |
|  | I can learn more about my patients’ cancer and treatment | **□** | **□** | **□** | **□** | **□** | **□** |
|  | I can better detect and manage my patients’ late effects | **□** | **□** | **□** | **□** | **□** | **□** |
|  | I have a schedule of the tests and examinations that my patients need | **□** | **□** | **□** | **□** | **□** | **□** |
|  | My communication with other **healthcare professionals** is improved | **□** | **□** | **□** | **□** | **□** | **□** |
|  | My **patients** know more about their cancer and treatment | **□** | **□** | **□** | **□** | **□** | **□** |
|  | My **patients** can better manage their late effects | **□** | **□** | **□** | **□** | **□** | **□** |
|  | My **patients** know more about the tests and examinations they need in the future | **□** | **□** | **□** | **□** | **□** | **□** |
|  | My **patients** feel less anxious or distressed about what will happen after their cancer treatment | **□** | **□** | **□** | **□** | **□** | **□** |
|  | My communication with patients is improved | **□** | **□** | **□** | **□** | **□** | **□** |
|  | They promote a healthy lifestyle to my **patients** | **□** | **□** | **□** | **□** | **□** | **□** |

**Healthcare providers’ perceived concerns about survivorship care plans**

1. **Please indicate how much you agree with the following statements. My workplace’s approach to survivorship care plans needs to be improved because:**

|  |  | **Strongly agree** | **Agree** | **Neither** | **Disagree** | **Strongly disagree** | **N/A** |
| --- | --- | --- | --- | --- | --- | --- | --- |
|  | I do not receive enough training about how to use survivorship care plans | **□** | **□** | **□** | **□** | **□** | **□** |
|  | I do not have enough time to create or deliver survivorship care plans | **□** | **□** | **□** | **□** | **□** | **□** |
|  | We do not have enough staff to create or deliver survivorship care plans | **□** | **□** | **□** | **□** | **□** | **□** |
|  | They do not contain enough information | **□** | **□** | **□** | **□** | **□** | **□** |
|  | They contain too much information | **□** | **□** | **□** | **□** | **□** | **□** |
|  | **Patients** find them difficult to understand or too complicated | **□** | **□** | **□** | **□** | **□** | **□** |
|  | They are not in a useful format (e.g. electronic or paper-based) | **□** | **□** | **□** | **□** | **□** | **□** |
|  | They are not given to **patients** at the right time | **□** | **□** | **□** | **□** | **□** | **□** |
|  | **Patients** do not use them | **□** | **□** | **□** | **□** | **□** | **□** |
|  | **Patients** feel more anxiety and distress | **□** | **□** | **□** | **□** | **□** | **□** |
